# Influenza A Virus on Oceanic Islands: Host and Viral Diversity in Seabirds in the Western Indian Ocean

**DOI:** 10.1371/journal.ppat.1004925

**Published:** 2015-05-21

**Authors:** Camille Lebarbenchon, Audrey Jaeger, Chris Feare, Matthieu Bastien, Muriel Dietrich, Christine Larose, Erwan Lagadec, Gérard Rocamora, Nirmal Shah, Hervé Pascalis, Thierry Boulinier, Matthieu Le Corre, David E. Stallknecht, Koussay Dellagi

**Affiliations:** 1 GIS CRVOI (Centre de Recherche et de Veille sur les maladies émergentes dans l'Océan Indien), Sainte Clotilde, Reunion Island; 2 Université de La Réunion, UMR PIMIT (Processus Infectieux en Milieu Insulaire Tropical), INSERM 1187, CNRS 9192, IRD 249, Saint Denis, Reunion Island; 3 Laboratoire d'Ecologie Marine, FRE 3560 INEE-CNRS, Université de La Réunion, Saint Denis, Reunion Island; 4 WildWings Bird Management, Grayswood Common, Haslemere, Surrey, United Kingdom; 5 Institut de Recherche pour le Développement, Sainte Clotilde, Reunion Island; 6 Island Conservation Society, Mahé, Seychelles; 7 Nature Seychelles, The Center for Environment and Education, Roche Caiman, Mahé, Seychelles; 8 Centre d’Écologie Fonctionnelle et Évolutive, Centre National de la Recherche Scientifique, Montpellier, France; 9 Southeastern Cooperative Wildlife Diseases Study, College of Veterinary Medicine, University of Georgia, Athens, Georgia, United States of America; Virginia-Maryland Regional College of Veterinary Medicine, UNITED STATES

## Abstract

Ducks and seabirds are natural hosts for influenza A viruses (IAV). On oceanic islands, the ecology of IAV could be affected by the relative diversity, abundance and density of seabirds and ducks. Seabirds are the most abundant and widespread avifauna in the Western Indian Ocean and, in this region, oceanic islands represent major breeding sites for a large diversity of potential IAV host species. Based on serological assays, we assessed the host range of IAV and the virus subtype diversity in terns of the islands of the Western Indian Ocean. We further investigated the spatial variation in virus transmission patterns between islands and identified the origin of circulating viruses using a molecular approach. Our findings indicate that terns represent a major host for IAV on oceanic islands, not only for seabird-related virus subtypes such as H16, but also for those commonly isolated in wild and domestic ducks (H3, H6, H9, H12 subtypes). We also identified strong species-associated variation in virus exposure that may be associated to differences in the ecology and behaviour of terns. We discuss the role of tern migrations in the spread of viruses to and between oceanic islands, in particular for the H2 and H9 IAV subtypes.

## Author Summary

Avian influenza viruses circulate in wild birds, worldwide, in particular in ducks and seabirds from which a large diversity of viruses have been described. The continued emergence of influenza viruses in poultry and humans has stimulated both research activities and surveillance programs; however, there are still many gaps in our knowledge on virus ecology and epidemiology, in particular in the Southern Hemisphere. In this study we investigated influenza virus circulation in seabirds in the islands of the Western Indian Ocean. We demonstrate that terns act as a major host for influenza viruses on oceanic islands and that, in addition to being infected with virus subtypes usually associated to wild birds, they also could regularly be in contact with viruses that represent a significant threat to veterinary and human health. This study demonstrates that the spatial isolation of these oceanic islands does not limit connectivity with the global avian influenza virus epidemiology and that it may create opportunities for local viral maintenance in wild bird communities.

## Introduction

Spatial isolation represents a major barrier to the introduction and transmission of infectious agents on oceanic islands. Animal migration is a key mechanism for the dispersal of infectious agents over long distances [1] and could play an important role in the spread of pathogens to island ecosystems. The introduction and spread of zoonotic diseases to and between oceanic islands is indeed likely to be closely associated to migratory movements of flying vertebrates such as birds and bats [2].

Wild birds are the reservoir for a large diversity of infectious agents that threaten human and veterinary health [3]. Ducks and seabirds are the natural hosts for avian influenza A virus (IAV) [4,5] and these hosts are the donors of gene segments and viruses that can eventually be responsible for outbreaks in livestock and humans [6]. The emergence of the H5N1, H7N9, and H9N2 virus subtypes in domestic birds in southeastern Asia, as well as the introduction of the swine-origin H1N1 virus in human populations, have demonstrated the ability of IAV to spread beyond species barriers and to adapt rapidly to new hosts and environmental conditions [7–9]. Over the past decade, ducks have been extensively studied and recognized as important hosts for IAV ecology. Other groups of birds, such as shorebirds and seabirds, are also often infected with IAV, but the epidemiological position that these hosts occupy between wild ducks, domestic birds and humans is not well understood [10].

On oceanic islands, species diversity, host abundance and density are generally higher for seabirds than for ducks, and is likely to affect the relative importance of these hosts in the ecology and evolution of IAV. In addition to spatial isolation, the small duck community size could limit opportunities for virus transmission and also negatively affect virus subtype diversity on these islands (ducks are the natural hosts for 14 of the 16 described hemagglutinin subtypes of avian IAV; [6]). Seabird migration and breeding behaviour could favour virus dispersal over long distances and transmission patterns within and between colonies. To date, most studies that have investigated virus circulation in seabirds have been conducted on continental and coastal habitats (see [10] for a review) and IAV epidemiology in seabird populations associated with oceanic islands has received little attention.

With an estimated breeding population size of 19 million individuals [11], seabirds represent the most abundant and widespread avifauna in the Western Indian Ocean [12]. The islands of this region are major breeding sites for terns (order Charadriiformes) as well as species in the orders Phaethontiformes (tropicbirds), Procellariformes (petrels and shearwaters), and Suliformes (boobies and frigatebirds). Several of these seabird species aggregate at very high densities in breeding colonies that may involve hundreds of thousands, occasionally millions, of birds [13], generating local conditions highly conductive for virus transmission. Because of their philopatry and high breeding-site fidelity, seabird populations are also highly structured in space [14]. This behavior could restrict virus exchanges between populations and species breeding on different islands or at different times of the year.

In this context, the first aim of this study was to identify the host range of avian IAVs in the seabird community of the Western Indian Ocean and to assess virus subtype diversity. Based on serological assays, we tested the effect of the bird order and species on the probability of detection of IAV antibodies on seven oceanic islands. We further tested a subset of samples for antibodies to specific HA subtypes and estimated the viral diversity of IAV circulating in terns. The second aim was to investigate the spatial variation in virus transmission between islands and identify the origin of circulating viruses. Molecular detection was carried out to assess the prevalence of infected birds and phylogenetic analyses were performed to identify the putative host and geographic origin of the detected viruses.

## Materials and Methods

### Ethic statement and research permits

The procedures performed in this study were not subjected to the approval of ethics committee neither to specific national or international regulations at the time of sample collection. Bird capture, handling and collection of biological material was nevertheless performed under research programs approved by the Center for Research on Bird Population Biology (Program 616; CRBPO; National Museum of Natural History, Paris). In addition, sample collection on Reunion and the Éparses Islands (Europa, Juan de Nova and Tromelin) were conducted under the approval of the “Direction de l'Environnement, de l'Aménagement et du Logement de la Réunion” and of the “Terres Australes and Antarctiques Françaises”. Bird capture, handling and collection of biological material in the Seychelles (Aride, Bird and Cousin Islands), as well as sample export to Reunion Island, were performed under the approval of the Seychelles Bureau of Standards and Department of Environment.

### Sample collection

**Fig 1:**
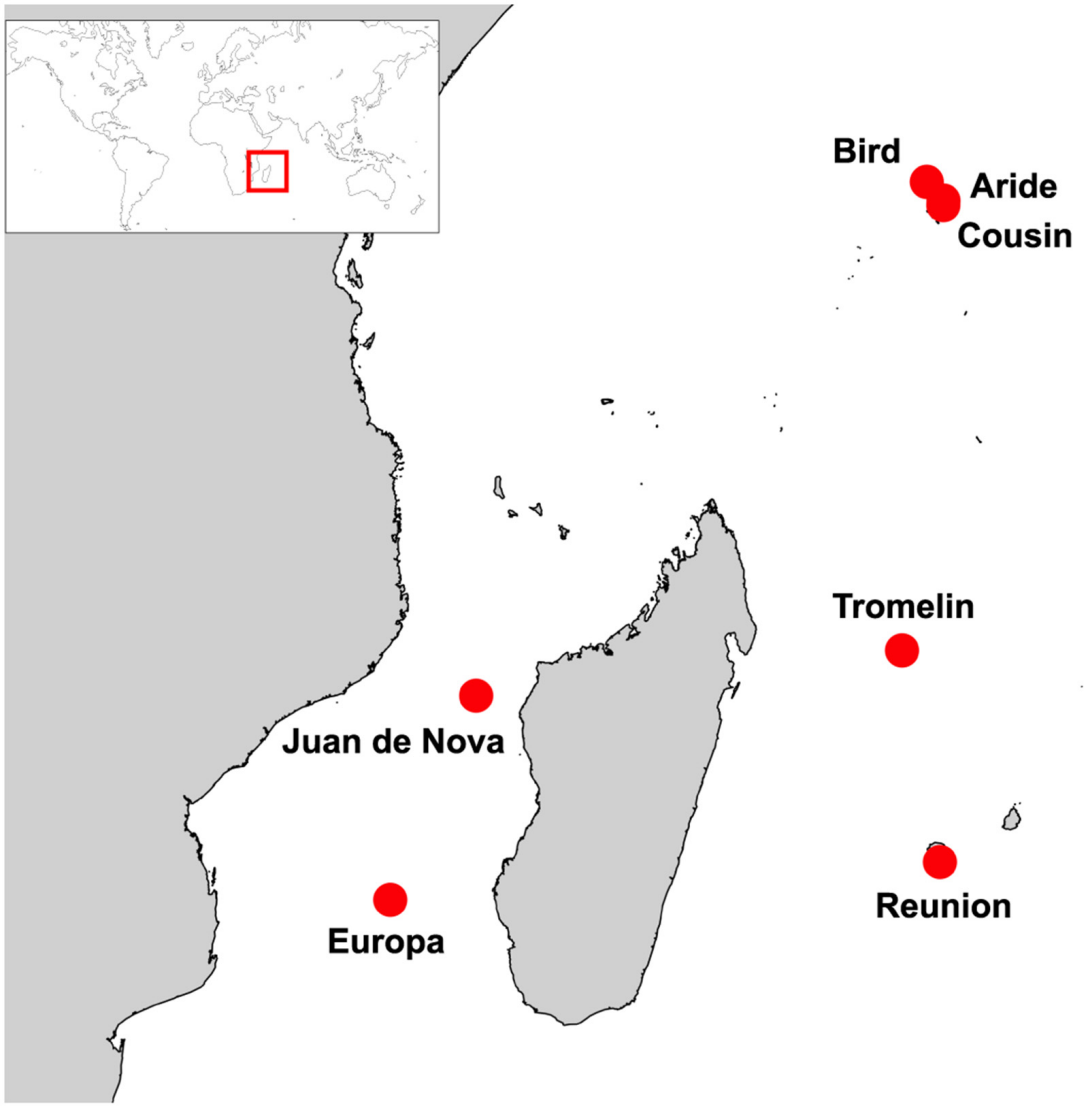
Sampling locations (red circles).

We focused on seven islands of the Western Indian Ocean (Fig 1): Aride (4°12'S, 55°39'E), Bird (3°43'S, 55°12'E), Cousin (4°19'S, 55°39'E), Europa (22°21'S, 40°21'E), Juan de Nova (17°03'S, 42°45'E), Reunion (21°22'S, 55°34'E), and Tromelin (15°53'S, 54°31'E). On these islands, seabird communities are highly heterogeneous in term of species richness, population size and density (S1 Table). To take into account this heterogeneity, our sampling strategy was designed to provide representative numbers of samples as a function of colony size and to include a maximum number of species on each island. For instance, eight seabird species breed on Europa, with population size ranging from ten to hundreds of thousands of breeding pairs (S1 Table; [15]). On this island, five of the eight breeding species were sampled and sample size was adjusted to be representative of the population size of each species (S2 Table). This sampling strategy was modified as needed related to local geographic, safety and ethical constraints that restrict access to bird colonies, such as in highly mountainous regions (*e*.*g*. on Reunion Island) or for species highly sensitive to human disturbance (*e*.*g*. frigatebirds).

Temporal variation in seabird breeding was also considered as it strongly affects the time of sample collection. In the tropics, seabirds species and populations do not breed at the same time of the year. For example, in the Western Indian Ocean, populations of sooty terns breed at different times on different islands. On Bird and Aride islands, breeding occurs during the austral winter (in June-July) while on Juan de Nova, it occurs during the austral summer (in December-January). Because of this temporal pattern, not all populations of the same species were sampled at the same time, and not all species of each island could be sampled during a single sampling session.

In total, 2997 samples (1647 sera and 1350 swabs) were collected from nine seabird species representing four different orders; Charadriiformes: Sooty tern, Brown noddy (*Anous stolidus*), Lesser noddy (*Anous tenuirostris*); Suliformes: Great frigatebird (*Fregata minor*), Masked booby (*Sula dactylatra*), Red-footed booby (*Sula sula*); Phaethontiformes: Red-tailed tropicbird (*Phaethon rubricauda*), White-tailed tropicbird (*Phaethon lepturus*); Procellariformes: Wedge-tailed shearwater (*Puffinus pacificus*). Details that relate to the number of collected samples for each species and island is presented in S2 and S3 Tables.

### Serology

A small sample of whole blood (maximum of 1.0% of body weight) was collected from the medial metatarsal or basilic veins, as appropriate for each species. In the field, blood samples were placed in 2 ml Eppendorf tubes in a cooler with ice packs and centrifuged within 12 hours after collection. Sera were transfered in cryotubes and stored at -20°C. Samples were shipped to the laboratory within 48 hours and held at -80°C until tested.

Serum samples were tested with the IDvet ID Screen Influenza A Antibody Competition (IDvet, Montpellier, France) enzyme-linked immunosorbent assay (ELISA), following an optimized protocol for the detection of antibodies to IAV nucleoprotein (NP) in wild birds [16]. Sample absorbance was measured at 450 nm with a Sunrise microplate reader (TECAN, Grödig, Austria). Samples with a sample-to-negative control ratio (S/N) below 0.4 were considered positive for the presence of IAV NP antibodies; samples with S/N greater than or equal to 0.55 were considered negative. Samples that yielded S/N between 0.4 and 0.55 were re-tested and, following the S/N obtained in the second test, were considered either negative (S/N > 0.4) or positive (S/N < 0.4).

Samples that tested positive for the presence of IAV NP antibodies with the ELISA were tested for the presence of HA-specific antibodies using virus neutralization (VN; for H1–H12, H14–H15 HA subtypes) and hemagglutination inhibition (HI; for H13, H16 HA subtypes) assays. Serum samples were heat-inactivated for 30 min at 60°C prior to the VN and HI assay. IAV strains used as antigens were: A/Mallard/NJ/AI10-4263/2010(H1N1), A/Mallard/MN/AI08-2755/2008(H2N3), A/Mallard/MN/AI10-2593/2010(H3N8), A/Mallard/MN/AI10-3208/2010(H4N6), A/Mallard/MN/AI11-3933/2011(H5N1), A/Mallard/MN/SG-00570/2008(H6N1), A/Mallard/MN/AI08-3770/2009(H7N9), A/Mallard/MN/SG-01048/2008(H8N4), A/Ruddy_turnstone/DE/AI11-809/2011(H9N2), A/Mallard/MN/SG-00999/2008(H10N7), A/Mallard/MN/SG-00930/2008(H11N9), A/Mallard/MN/SG-3285/2007(H12N5), A/Ringed-billed gull/MN/AI10-1823/2010(H13N6), A/Blue-winged teal/TX/AI13-1028/2013(H14N5), A/Wedge-tailed_shearwater/Western_Australia/2327/1983(H15N6), A/Ringed-billed gull/DE/AI10-1144/2010(H16N3). The HI assay was done as described in [17].

For the VN assay, 25 μL of sample diluted 1:10 in minimal essential medium (MEM) supplemented with antibiotics (10000 U/ml penicillin G, 10 mg/ml streptomycin, and 25 mg/ml amphotericin) were placed in a 96-well plastic V-bottomed microtitre plate. Twenty-five microlitres of virus suspension (100 median tissue culture infectious dose (TCID_50_) per 25 μL) diluted in MEM supplemented with antibiotics and TPCK-trypsin (final concentration of 1 μg/ml; Worthington Biochemical Corporation, Lakewood, New Jersey, USA) were added in each well and the plate was incubated at room temperature for two hours. After incubation, 25 μl from each well were transferred to a 96-well cell culture plate with a confluent monolayer of Maden Darby canine kidney (MDCK) cells (ATCC, Manassas, Virginia, USA) and incubated for 3 days at 37°C under 5% CO_2_. Prior to transfer, MDCK plates were washed twice with DPBS (150 μL per well) and media in each well was replaced with 150 μL of trypsin-supplemented MEM (as described above). Examination for cytopathic effects was performed with light microscopy; samples were considered positive when no cytopathic effect was observed. A single well without antigen was included for each sample to test for toxicity. In addition, a positive and negative well (MEM only) was run for each antigen and all antigens were back titrated to confirm the proper dilution. Positive reference antisera for the 16 HA subtypes were provided by National Veterinary Services Laboratory, APHIS, USDA.

All samples testing positive at the 1:20 dilution were tested again at dilutions ranging from 1:20 to 1:640. Fifty microlitres of each previously diluted sample (1:10) was placed in the first line of a 96-well plastic V-bottomed microtitre plate and serially diluted in 25 μl of trypsin supplemented MEM. Antigen (25 μl) was then added in each well and the plate was incubated as described before; all previously described controls and back-titrations were also performed. The VN titre was considered to be the highest dilution without cytopathic effects and if testing was negative at the 1:20 dilution the samples were considered negative.

### Statistical analyses

Generalized linear models (GLMs) with a binomial error structure were used to examine the effect of the island, bird order, time of sampling and age (adult *versus* chick), on the probability of successful detection IAV NP antibodies in bird serum. GLMs were also used to test the effect of the bird species and island on the probability of successful detection of IAV NP antibodies specifically in Charadriiformes. A second-order Akaike information criterion (AIC) was used to select the most parsimonious model. The effect of variables included in the most parsimonious model was tested using a Chi square test (χ^2^). Analyses were conducted in R 2.15.2 [18].

### Molecular detection and sequencing

Cloacal and oropharyngeal swab samples were obtained using sterile cotton tipped applicators. Samples were placed in 1 ml of RNA NOW (BIOGENTEX, Seabrook, Texas, USA) except for samples collected in 2013 that were placed in 1 ml of Brain Heart Infusion (BHI) media (Conda, Madrid, Spain) supplemented with penicillin G (1000 units/ml), streptomycin (1 mg/ml), kanamycin (0.5 mg/ml), gentamicin (0.25 mg/ml), and amphotericin B (0.025 mg/ml). Swabs were maintained in a cooler with ice packs in the field and stored at -20°C within 12 hours. Samples were shipped to the laboratory within 48 hours and held at -80°C until tested.

Tubes containing cloacal and oropharyngeal swabs were vortexed and centrifuged at 1500 g for 15 min. RNA extraction was performed following RNA NOW isolation and purification protocol, or with the QIAamp Viral RNA Mini Kit (QIAGEN, Valencia, California, USA) for samples stored in BHI supplemented with antibiotics. Reverse-transcription was performed following a previously published protocol [19]; cDNA were diluted 1:2 and tested for the presence of IAV Matrix (M) RNA by real-time polymerase chain reaction (rt-PCR) [20]. All rt-PCR positive samples were confirmed by sequencing the amplicons (Genoscreen, Lille, France).

Subtyping of the positive samples was attempted by PCR using primer sets designed for the amplification of avian HA (H1–H16) and NA (N1–N9) IAV subtypes [20–24]. Amplification products were analyzed by electrophoresis on a 2% agarose gel stained with 0.4% GelRed (Biotum, Hayward, California, USA). Partial sequencing was performed by Genoscreen.

### Phylogenetic analyses

A preliminary phylogenetic analysis was performed with all IAV H2 HA nucleotide sequences available in the Influenza Sequence Database ([25]; N = 618). Briefly, the coding region of nucleotide sequences was aligned with CLC 6.6.2 (CLC bio, Aarhus, Denmark). Maximum-likelihood analyses were performed using the software PhyML 3.1 [26], with the general time reversible (GTR) evolutionary model, an estimation of the proportion of invariable sites (I) and of the nucleotide heterogeneity of substitution rates (α). The phylogenetic tree obtained from this preliminary analysis is presented in S1 Fig (the detailed phylogenetic tree is available upon request).

A Bayesian Markov Chain Monte Carlo coalescent analysis was then conducted to investigate the recent evolutionary history of the genetic lineage that included Reunion Island viruses (red branches on S1 Fig; N = 106). Analyses were performed with the program BEAST 1.7.4 [27]; overall, a similar methodology was used as in previous studies on IAV evolutionary dynamics in wild birds [28,29]. The uncorrelated exponential molecular clock was selected following Bayes Factors comparison with estimates obtained with the strict clock and uncorrelated lognormal clocks. The SRD06 nucleotide substitution model [30] and a Bayesian skyline coalescent tree prior were used in all simulations [31]. Two independent analyses were performed with a chain length of 100 million generations sampled every 1000 iterations. Analyses were combined after the removal of an appropriate burn-in (10% of the sampled trees).

## Results

### Host diversity and spatial distribution

**Fig 2:**
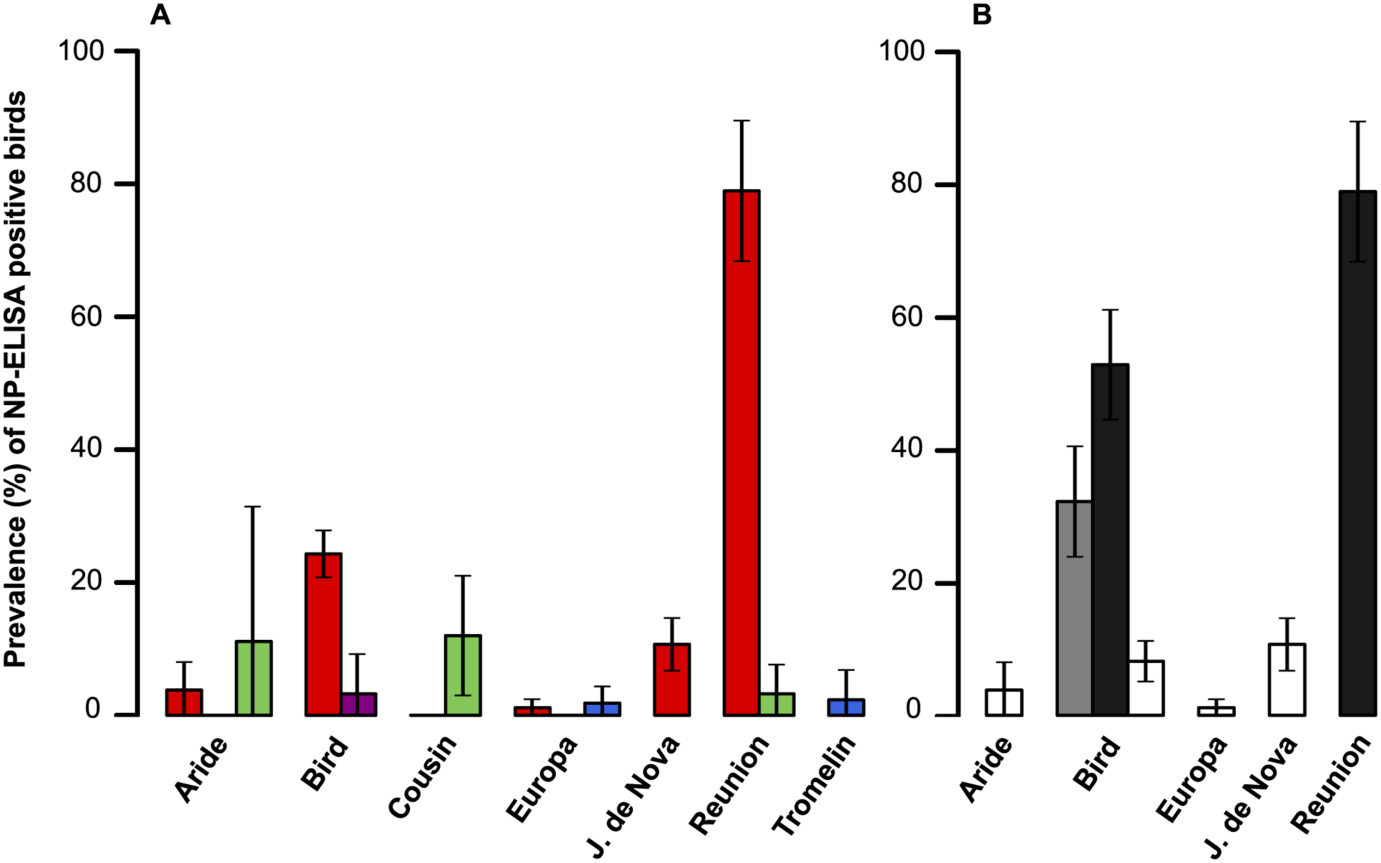
(A) Influenza A virus host diversity. Prevalence of seropositive samples (percentage with 95% confidence interval), for each island and bird order (red: Charadriiformes; green: Procellariformes; purple: Phaethontiformes; blue: Suliformes). (B) Influenza A virus host diversity in birds in the Charadriiformes order. Prevalence of seropositive samples (percentage with 95% confidence interval), for each island and bird species (black: Lesser noddy; gray: Brown noddy; white: Sooty tern).

A total of 1647 sera were collected on seven islands of the Western Indian Ocean (S2 Table). Overall, 227 samples (13.8%) tested positive for the presence of IAV NP antibodies; details on sampled species, locations, collection date and bird status are presented in S2 Table. The probability of detection of IAV NP antibodies was significantly different among bird orders (GLM with binomial errors; χ^2^ = 89.7, *p* < 0.001). The mean prevalence of seropositive birds ± 95% confidence interval was: Charadriiformes: 17.8 ± 2.2%; Procellariformes: 7.4 ± 4.7%; Suliformes: 1.9 ± 2.2%; Phaethontiformes: 0.6 ± 1.1%). A strong island effect was also found (χ^2^ = 210, *p* < 0.001; Fig 2a), as well as an effect of the time of sampling (χ^2^ = 301, *p* < 0.001). Finally, the most parsimonious model identified with the AIC did not included the bird age; this finding was likely affected (*i*) by the low number of sampled chicks, and (*ii*) by the species and population in which chicks were sampled that corresponded mainly to those with low prevalence or absence of seropositive adults.

When only Charadriiformes were included in the analysis (Fig 2b), the probability of detection of IAV NP antibodies varied significantly between species (χ^2^ = 292, *p* < 0.001; Lesser noddy: 60.4 ± 6.8%; Brown noddy: 32.2 ± 8.3%; Sooty tern: 6.35 ± 1.6%), and between islands (χ^2^ = 219, *p* < 0.001). Differences in the prevalence of seropositive birds were also detected between bird populations of the same species. The four sampled Sooty tern colonies had significantly different prevalences (χ^2^ = 26.5, *p* < 0.001), with higher prevalences found on Juan de Nova (10.7 ± 4.0%) and Bird Island (8.1 ± 3.1%) as compared to Aride Island (3.8 ± 4.2%) and Europa (1.2 ± 1.3%). We also found a significant difference between the two populations of lesser noddies (χ² = 12.2, *p* < 0.001), with a higher prevalence of seropositive birds on Reunion Island (78.9 ± 10.6%) than on Bird Island (52.9 ± 8.3%). On Bird Island, lesser noddies, brown noddies and sooty terns were sampled both in June 2012 and June 2013, but no significant variation in the prevalence of seropositive birds was found between years (χ^2^ = 1.01, *p* = 0.32).

### Virus subtype diversity

Of the 227 samples that tested ELISA positive, 156 (67%) were further tested for the identification of HA-specific antibodies with the VN or HI assays. The remaining 71 samples could not be tested because of the limited volume of serum available. For the same reason, not all samples were tested for all 16 described IAV HA subtypes. In total, 147 samples (94%) were tested for H1–H9, H11–H12 and H14–H15, 103 (66%) were tested for H10, and 88 (56%) for H13 and H16.

**Fig 3:**
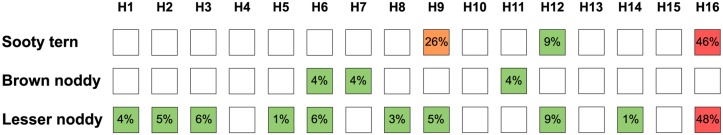
Hemagglutinin (HA)-specific antibody diversity in species in the Charadriiformes order. Numbers indicate the proportion of sample that tested positive for each subtype, based on the total number of samples tested for the same subtype. For instance, for sooty terns, 9 out of the 35 tested serum samples (26%) tested positive for the detection of H9 antibodies. Detailed results are presented in S4 Table.

Of the positive samples, HA-specific antibodies were detected in fifty-nine samples (38%). The HA subtype diversity was higher in lesser noddies than in brown noddies and sooty terns, with 10 out of the 16 described avian HA subtypes detected in lesser noddies (Fig 3; S4 Table). H16 was the most commonly detected subtype although H9 also was common in sooty terns, with 9 occurrences out of 19 samples (47%) that tested positive with the VN and HI assays. In most cases, antibodies reacted with only one HA subtype; however, multiple HA subtypes were also detected. Hence, four samples from sooty terns tested positive for more than one HA subtype, with combinations of H16 and H9, or H16 and H12, but not H9 and H12. The presence of multiple HA-subtypes was also frequent in lesser noddies (13 out of 38 samples; 34%), with either combinations between H16 and H1, H3, H6, H9 or between H12 and H1, H2, H3, H5, H6, H8, H9; only one sample tested positive for antibodies against both the H12 and H16 subtypes. For brown noddies, HA-specific antibodies were detected in only two out of the 26 samples that tested positive with the ELISA; one of the two samples tested positive for both the H7 and H11 HA subtype. None of the ELISA positive samples collected in non-Charadriiformes species tested positive with the VN and HI assays (S4 Table).

### Virus detection

Seventeen out of the 1350 cloacal (N = 1086) and oropharyngeal (N = 264) swab samples tested positive for the presence of IAV M RNA by rt-PCR (S3 Table). All positive samples were obtained from non-breeding lesser noddies, on Reunion Island. Fourteen cloacal and three oropharyngeal swabs collected in these birds tested positive; only one of the 58 tested birds was excreting viruses in both the cloaca and the oropharyngeal cavity at the time of sample collection. The prevalence (± 95% confidence interval) of virus shedding in lesser noddies on Reunion Island was 27.6 ± 11.5%. When HA and NA subtyping was performed on the positive samples from Reunion Island, nine samples tested positive for the H2 HA subtype. The HA of the remaining samples could not be determined but this negative result was most likely due to the very small amount of RNA present in the sample rather than reflecting shedding of a different HA subtype in the studied population. We indeed found that the mean cycle threshold of the IAV M rt-PCR was significantly higher for samples for which HA subtyping failed than for samples for which H2 subtype were identified (t = 4.74, df = 14.7, *p* < 0.001). Finally, although PCRs were carried out for the nine described avian NA subtypes, we could not identify the NA subtype of Reunion Island viruses.

**Fig 4:**
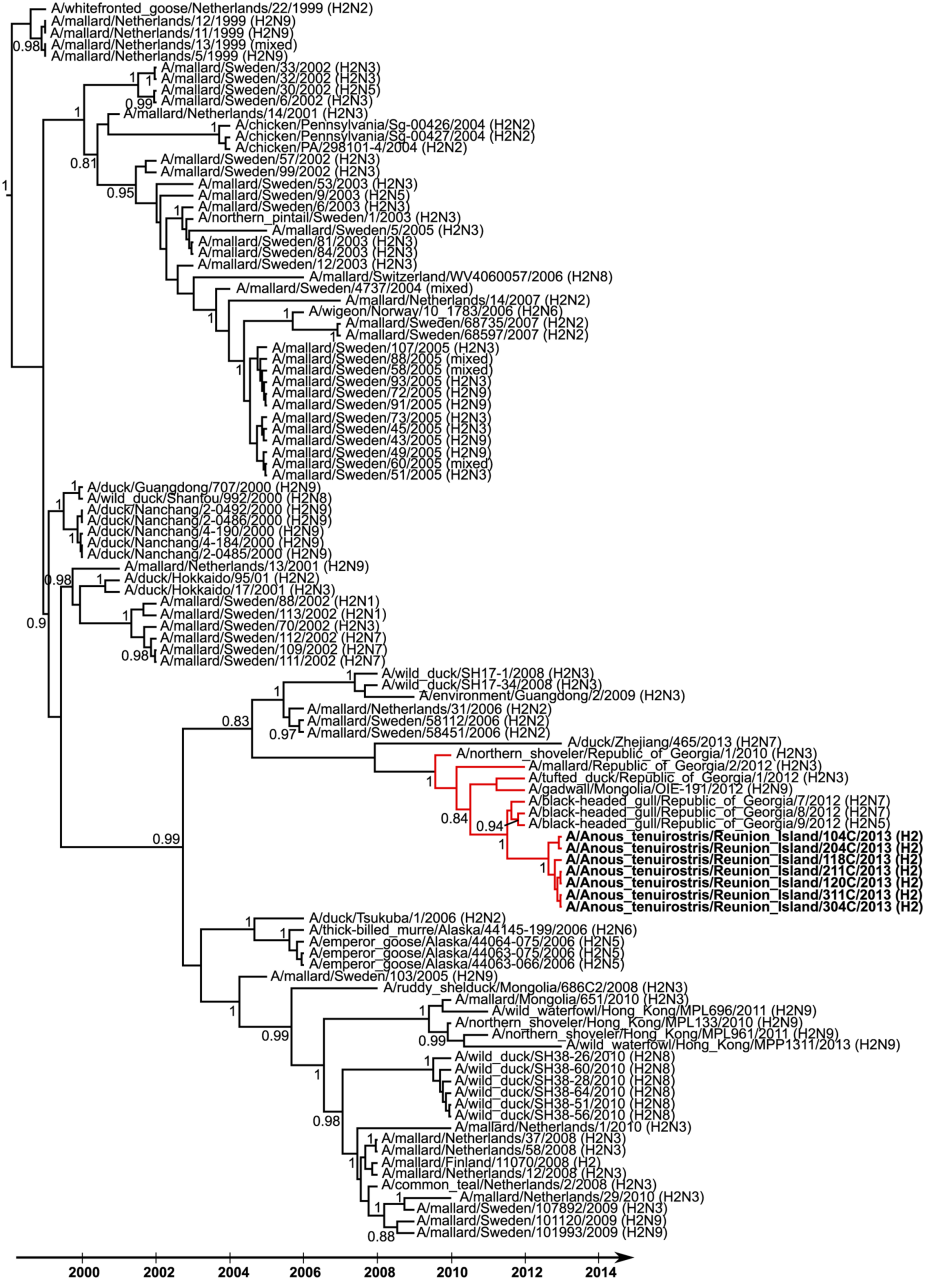
Maximum clade credibility tree derived from 106 influenza A virus H2 hemagglutinin nucleotide sequences (908 bp). Posterior probability higher than 0.8 are reported. Reunion Island viruses are indicated in bold. Red branches indicate the genetic lineage including the most recent ancestors of the Reunion Island viruses.

Partial nucleotide sequences of HA of seven H2 IAV detected on Reunion Island were obtained (sequence lengths of either 910 bp or 1013 bp). The limited genetic diversity observed (nucleotide sequence similarity ranging from 99.8% to 100%) indicate that a single H2 IAV subtype was likely circulating at the time of bird sampling. Phylogenetic analyses suggested that these viruses were closely related to those that have been circulating in wild birds in Eurasia since 2009 (time of the most recent common ancestor [± 95% HPD]: 2009.6 [2008.6–2010]; Red lineage on Fig 4). In particular, the Reunion Island viruses were closely related to H2N5 and H2N7 IAV isolated in black-headed gulls (*Chroicocephalus ridibundus*) in the Republic of Georgia, in 2012. The time of the most recent common ancestor was 2011.5 [2011–2011.9], suggesting recent gene flow between Eurasia and the Southwestern Indian Ocean.

## Discussion

Based on serological assays, we identified the host range of IAV in seabirds, the most abundant and widespread avifauna of the Western Indian Ocean, and further assessed the virus subtype diversity in terns. Our findings strongly support that terns, in particular brown and lesser noddies, represent the major hosts for IAV in this region. In contrast, species of the orders Phaethontiformes, Procellariformes and Suliformes may have a more limited role in IAV epidemiology on the islands of the Western Indian Ocean. Our results indicate that terns host not only seabird-associated virus subtypes such as H16, but that they can be infected by viruses commonly isolated from wild ducks (H3, H6, H12 subtypes), and IAV subtypes that represent a significant threat to veterinary and human health (H1, H5, H7 and H9).

Several limitations in our study need nevertheless to be taken into consideration. Firstly, although our sampling strategy was designed to provide representative numbers of samples as a function of colony size and from a maximum number of species, a subset of seabird species and populations were included in our analysis and one cannot exclude that other species may also be actively involved in IAV epidemiology on oceanic islands (*e*.*g*. shorebirds). Secondly, as North American IAV isolates were used as reference viruses in the VN and HI assays (except for H15), we cannot exclude that they may be antigenically different to IAV circulating in the Western Indian Ocean, and this may have resulted in underestimating subtype prevalence. Thirdly, failure to isolate viruses from positive samples precluded the sequencing of the full genomes of the H2 IAVs from Reunion Island, therefore limiting possible inferences on their phylogenetic and geographic origins.

The first isolation of an IAV in wild birds was reported in 1961 following an epizootic among common terns (*Sterna hirundo*) that occurred in South Africa [32]. Although the origin of the highly pathogenic H5N3 virus responsible for the death of at least 1300 common terns remains unknown, this event suggested that terns could play an important role in the global ecology and epidemiology of IAV in the Southern hemisphere [32]. In the late 1970's, IAV were also isolated in sooty terns and lesser noddies on Pelsart Island, Western Australia [33], where the H15 virus subtype was first reported [34].

The global prevalences of infected (0.9%; [6]) and seropositive birds (1.3%; [35]) are extremely low in terns, especially when compared to wild ducks and gulls. Nevertheless, our study indicates that extensive species-related variation could exist in terns with regard to IAV exposure. The high seroprevalence that we detected in brown and lesser noddies (32.2% and 60.4%, respectively) was comparable to antibody prevalence estimates for Mallard (*Anas platyrhynchos*) and other wild duck species that are recognized as natural host reservoirs for IAV (*e*.*g*. 46% in mallards in North America; [35]). This result suggests that virus exposure varies greatly between species and that some species, such as those of the genus *Anous* (brown and lesser noddies), may play a central role in the epidemiology of IAV on oceanic islands.

Particularities of the ecology of terns might account for the variation in virus exposure and transmission between closely related species. Species-related differences in life history such as colonial nesting, social behaviour, migration and foraging characteristics are indeed likely to affect opportunities for virus transmission. For instance, Sooty terns breed in colonies of hundreds of thousands of pairs, with high bird densities [13], potentially generating optimal conditions for virus transmission. Yet, in our study, IAV were not directly detected and the seroprevalence was relatively low in these breeding colonies which in some cases were composed of more than a million pairs and very high nest densities (*e*.*g*. six nests per square meter on Bird Island [36]). A potential relationship between nest density and seroprevalence might nevertheless exist because higher prevalences were measured on Bird Island and Juan de Nova, where nest density was higher than on Aride and Europa (less than two nests per square meter). This potential trend needs to be validated by taking into account inter-annual variation as well as intra-colony differences in nest density.

Population size and density may not be the only ecological drivers for IAV transmission in tern colonies and other life history characteristics, such as behaviour during the post-breeding migration, could play a more significant role. Sooty terns remain at-sea without land stop-over during the non-breeding period. In contrast, brown and lesser noddies tend to visit and roost on a large number of islands in the Indian Ocean after the breeding season [37]. These post-breeding roosting sites may favor high contact rates within and between species, which could favor virus transmission. Although further research is necessary on these aspects, our findings indicate that differences in the ecology and behaviour of closely related tern species could significantly affect virus exposure and transmission opportunities. Such a pattern would thus be similar to the differences that are reported between closely related ducks species, in particular between dabbling and diving ducks [6].

Spatial and temporal variation in virus transmission may also exist between tern populations of the same species. This is suggested by the significant differences in seroprevalences estimated at different time of the year, in the populations of lesser noddies sampled on Bird and Reunion islands. In a recent study, Verhagen *et al*. reported very high prevalence of infections (up to 72%) in nesting and fledging black-headed gulls [38]. The authors did not detect virus in the adults they sampled throughout the course of the breeding season, but seroprevalence in these birds reached about 40% [38]. In our study, higher seroprevalence was found in lesser noddies on Reunion Island (78.9 ± 10.6%), where sampling was conducted in a roosting site during the post-breeding migration, than on Bird Island (52.9 ± 8.3%), where sampling was performed during the breeding season. This pattern suggests that, in addition to spatial variation, a strong temporal variation associated to the host life cycle may affect virus transmission opportunities. The high prevalence of infection we report on Reunion Island (27.6 ± 11.5%) highlights that transmission may occur preferentially on roosting sites rather than on breeding colonies and further supports that significant temporal as well as age-associated variations in virus infection could exist for seabirds.

Our findings stress that terns are exposed to a high diversity of IAV subtypes, and that this diversity differs between species. Similarly to gulls [39], noddies may represent important mixing vessels for IAV and favor genetic reassortment between viruses circulating in a large diversity of hosts and geographic areas. In wild ducks, co-infections with different virus subtypes frequently occur [40], resulting in high rates of genetic reassortment without clear patterns of gene segment association or virus fitness costs [41–43]. In gulls, high intercontinental virus gene exchange has been reported suggesting that seabirds could represent important vectors for geographically reassorted viruses [39]. It remains unknown whether co-infection frequently occurs in noddies, or if the detection of antibodies against multiple IAV subtypes results from sequential infections. In parallel, the consequences of genetic reassortment between seabird-, duck-, and poultry-associated viruses related to viral shedding, transmission and maintenance in the environment, require further investigations.

In both sooty terns and lesser noddies, H16 was the most common subtype, supporting that, together with gulls, terns could be a major host reservoir for this virus subtype. None of the samples that tested ELISA positive for antibodies tested positive with the HI assay for the H13 HA subtype. As previously mentioned, we cannot exclude that this negative result merely reflects a technical limitation with our HI assay because of significant antigenic differences between the North American origin H13 IAV used in the serological assays and H13 IAV circulating in the Indian Ocean.

The variations reported in the detected subtypes as well as in their number also reflects significant species-related differences in virus transmission. As compared to sooty terns, noddies may be more frequently exposed to a wider diversity of IAV as result of gregarious behaviour and mixing with conspecific and interspecific individuals during the post-breeding season. The detection of IAV subtypes usually associated with wild ducks and poultry (H3, H6, H9, H12) further suggests that noddies might share habitats with ducks at some stage during their life cycle. Mixing between species and populations originating from different geographic areas favour virus gene flow between hosts and facilitates cross-species transmission, in particular during the course of migration [44,45] and, for pelagic birds, in wintering areas [46,47]. The variation observed between sooty terns and noddies could thus be due to differences in species and population mixing during the post-breeding migration.

In the Western Indian Ocean, however, the absence of indigenous duck species on oceanic islands and the limited influx of migrant ducks [12], potentially restrict host shifts opportunities. Dabbling ducks may maintain IAV transmission but this could be limited to Madagascar and to the coast of Africa. Among the surface-feeding duck species inhabiting Madagascar, the Red-billed teal (*Anas erythrorhyncha*) may for instance be involved in local IAV circulation [48]. The Garganey (*Anas querquedula*) is also present on the eastern coast of Africa and is an occasional non-breeding visitor to several Western Indian Ocean islands [12]. This long-distance migrant is a major host for IAV [49–52]; one could thus hypothesize that it may be involved in intercontinental virus gene flow from the Northern to the Southern hemisphere. In addition to ducks, the role of shorebirds in the dispersal of IAV along the coast of Africa also remains to be assessed as these long-distance migrants have been identified as important hosts in other regions [45,53].

The detection of the H2 IAV on Reunion Island further supports that gene flow between Eurasia and the Western Indian Ocean can occur. Genetic analyses indeed showed that the virus detected on Reunion Island was closely related to H2 IAV isolated in black-headed gulls in the Republic of Georgia, and suggested that this virus might have been introduced to the Western Indian Ocean by long-distance migrants in the East-Asia—East-Africa or Black Sea—Mediterranean migratory flyway. Overlap between the spatial ranges of black-headed gulls and noddies could have favoured direct virus transmission of this virus subtype, usually isolated in ducks. The limited information on IAV circulation in wild birds in Eastern Africa, as well as the limited knowledge on seabird migrations in this area, however, precludes a more precise conclusion on the geographic and host origins of the Reunion Island viruses.

The high detection rate of H9 HA-specific antibodies in sooty terns (*e*.*g*. 40% of the birds that tested positive for the presence of IAV NP antibodies on Bird Island) indicates that this species is regularly in contact with this IAV subtype. The H9 IAV subtype is relatively uncommon in wild ducks [54,55] and has occasionally been found in seabirds and shorebirds (*e*.*g*. [56,57]). The H9N2 virus subtype has been circulating in poultry in Asia since the mid-1990s [58] and was identified as being the donor of the internal segments of the Asian strains of the H5N1 and H7N9 viruses [9,59]. This virus subtype has also been responsible for infections in humans [60], and has been reported as the predominant virus subtype in poultry and live-bird markets, between 2008 and 2011 in Bangladesh [61,62] and in 2003 and 2004 in India [63]. Interestingly, recent studies have revealed that sooty terns from Bird Island spend long periods of time in the Bay of Bengal during their post-breeding migration [64]. Although it remains unclear if prolonged activities of sooty terns in this region could have led to infection with H9N2 viruses circulating in poultry, this finding nevertheless supports that terns may regularly be infected with virus subtypes that are considered at threat to veterinary and human health. Future isolation and genetic analyses of H9 IAV subtypes infecting sooty terns will provide precise information on potential gene flows between Southern Asia and the Western Indian Ocean.

Our study stresses the potential role of seabird migrations and behaviour during the non-breeding period in the spread of IAV on oceanic islands. We highlight that the spatial isolation of oceanic islands is unlikely to disconnect virus transmission from the global IAV epidemiology and that it may create opportunities for their local maintenance in wild bird communities. Future investigations will have to focus specifically on tern population structure and migratory pattern to better assess the risk associated to virus transmission within and between islands.

## Supporting Information

S1 Table General information on the studied islands and seabird communities. Numbers are expressed in breeding pairs.
(PDF)

S2 Table Location, species, date of collection, bird status and number (N) of tested and positive serum samples for the presence of influenza A virus nucleoprotein antibodies. (PDF)

S3 Table Location, species, date of collection, bird status and number (N) of tested and positive cloacal (CL) and oropharyngeal (OP) swab samples for the presence of influenza A virus Matrix RNA. (PDF)

S4 Table List of the 156 serum samples tested by virus neutralisation (H1–H12 and H14–H15 subtypes) and hemagglutination inhibition (H13 and H16 subtypes) assays. Titres are indicated for each tested sample and highlighted in red when considered positive. NT: not tested.
(PDF)

S1 Fig Maximum likelihood consensus cladogram derived from 618 influenza A virus H2 hemagglutinin nucleotide sequences. Computations were realized with the GTR+I+ Γ evolutionary model (I = 0.3; α = 1.3). Blue branches highlight viruses isolated in humans and green branches viruses recovered from birds, swines and from the environment. Red branches highlight the genetic lineage of Reunion Island H2 influenza A viruses for which the detailed evolutionary history was investigated with coalescent analyses (Fig 4). Bootstrap values are indicated for the main phylogenetic lineages (black circles).
(PDF)
