# Supplementary material for: Influenza A Virus on Oceanic Islands: Host and Viral Diversity in Seabirds in the Western Indian Ocean
Source: PLoS Pathog. 2015 May 21;11(5):e1004925. doi: 10.1371/journal.ppat.1004925 (PMC4440776; doi:10.1371/journal.ppat.1004925)
Supplement: S1 Table — Numbers are expressed in breeding pairs. (PDF) [file ppat.1004925.s001.pdf]

Bird species	Aride	Bird	Cousin	Europa	Juan de Nova	Reunion	Tromelin
Charadriiformes							
Bridled tern (*Sterna anaethetus*)	70-200		580[e]				
Brown noddy (*Anous stolidus*)	1700-10400	10000[b]	1700[e]			300	
Caspian tern (*Hydropogne caspia*)				10			
Crested tern (*Thalasseus bergii*)					250		
Lesser noddy (*Anous tenuirostris*)	93000-193000	9000[c]	71900-90000[d]				
Roseate tern (*Sterna dougalii*)	300-1650						
Sooty tern (*Onychoprion fuscatus*)	156000-437000	500000[a]		760000	2000000		
White tern (*Gygis alba*)	5300-10400	500[a]	1860-4080[d]				3
Suliformes							
Greater frigatebird (*Fregata minor*)	3000-4500*			1100			
Lesser frigatebird (*Fregata ariel*)				1200			
Brown nooby (*Sula leucogaster*)							1
Masked booby (*Sula dactylatra*)							1090
Red-footed booby (*Sula sula*)				3000			855
Phaethontiformes							
Red-tailed tropicbird (*Phaethon rubricauda*)	2-5			3500			
White-tailed tropicbird (*Phaethon lepturus*)	600-1500	30[a]	860-1540[d]	1000		2020	
Procelariformes							
Wedge-tailed shearwater (*Puffinus pacificus*)	19500	500[a]	9250-16900[d]			1000	
Tropical shearwater (*Puffinus lherminieri*)	57000		7550[e]	50		3000	
Mascarene petrel (*Pseudobulweria aterrima*)						50	
Barau's petrel (*Pterodroma baraui*)						5000	
References	[1]	[a] [2] ; [b] [3] ; [c] [4]	[d] [5] ; [e] [6]	[7]	[7]	[6]	[8]

* includes both Frigatebird species.

References

1. Bowler J, Betts M, Bullock I, Ramos JA. Trends in seabird numbers on Aride Island Nature Reserve, Seychelles 1988-2000. Waterbirds. 2002;25: 26-38.
2. Feare CJ. Unpublished data. 2014.
3. Feare CJ. The ecology of Bird Island, Seychelles. Atoll Res Bull. 1979;226: 1-29.
4. Bristol R. Unpublished data. 2002.
5. Rocamora G, Skerrett A. Seychelles. In Fishpool LDC, Evans MI (editors). Important bird areas in Africa and associated islands. Cambridge, UK: BirdLife International. 2001; 751-768.
6. Le Corre M. Unpublished data. 2007.
7. Le Corre M, Jaquemet S. Assessment of the seabird community of the Mozambique Channel and its potential use as an indicator of tuna abundance. Estuar Coast Shelf Sci. 2005;63: 421-428.
8. Le Corre M, Danckwerts DK, Ringler D, Bastien M, Orlowski S, Rubio CM, et al. Seabird recovery and vegetation dynamics after Norway rat eradication at Tromelin Island, Western Indian Ocean. Biol Cons. 2015;185: 85-94.
