# Supplementary material for: Influenza A Virus on Oceanic Islands: Host and Viral Diversity in Seabirds in the Western Indian Ocean
Source: PLoS Pathog. 2015 May 21;11(5):e1004925. doi: 10.1371/journal.ppat.1004925 (PMC4440776; doi:10.1371/journal.ppat.1004925)
Supplement: S2 Table — (PDF) [file ppat.1004925.s002.pdf]

Island	Species		Date	Status	N tested	N Positive (%)
Aride	Sooty tern	*Onychoprion fuscatus*	Jun. 2012	Breeding adults	79	3 (3.8)
	White-tailed tropicbird	*Phaethon lepturus*	Jun. 2012	Breeding adults and chicks	14	0
	Wedge-tailed shearwater	*Puffinus pacificus*	Jun. 2012	Breeding adults	9	1 (11.1)
Bird	Brown noddy	*Anous stolidus*	Jun. 2012	Breeding adults	31	11 (35.5)
	Lesser noddy	*Anous tenuirostris*	Jun. 2012	Breeding adults	50	23 (46)
	Sooty tern	*Onychoprion fuscatus*	Jun. 2012	Breeding adults	207	15 (7.2)
	Brown noddy	*Anous stolidus*	Jun. 2013	Breeding adults	90	28 (31.1)
	Lesser noddy	*Anous tenuirostris*	Jun. 2013	Breeding adults	90	51 (56.7)
	Sooty tern	*Onychoprion fuscatus*	Jun. 2013	Breeding adults	100	10 (10)
	White-tailed tropicbird	*Phaethon lepturus*	Jun. 2012	Breeding adults and chicks	31	1 (3.2)
Cousin	White-tailed tropicbird	*Phaethon lepturus*	Nov. 2011	Breeding adults and chicks	43	0
	Wedge-tailed shearwater	*Puffinus pacificus*	Nov. 2011	Breeding adults	50	6 (12)
Europa	Great frigatebird	*Fregata minor*	Dec. 2011	Chicks	18	1 (5.6)
	White-tailed tropicbird	*Phaethon lepturus*	Dec. 2011	Breeding adults	29	0
	Red-tailed tropicbird	*Phaethon rubricauda*	Dec. 2011	Breeding adults	45	0
	Red-footed boody	*Sula sula*	Dec. 2011	Nonbreeding adults	30	0
	Sooty tern	*Onychoprion fuscatus*	Jul. 2012	Breeding adults	184	2 (1.1)
	White-tailed tropicbird	*Phaethon lepturus*	Jul. 2012	Breeding adults	12	0
	Great frigatebird	*Fregata minor*	Nov. 2012	Breeding adults and chicks	25	1 (4)
	Sooty tern	*Onychoprion fuscatus*	Nov. 2012	Breeding adults and chicks	78	1 (1.3)
	Red-footed boody	*Sula sula*	Nov. 2012	Breeding adults and chicks	36	0
Juan de Nova	Sooty tern	*Onychoprion fuscatus*	Dec. 2012	Breeding adults and chicks	234	25 (10.7)
Reunion	Wedge-tailed shearwater	*Puffinus pacificus*	Nov. 2011	Breeding adults	25	0
	Wedge-tailed shearwater	*Puffinus pacificus*	Dec. 2012	Breeding adults	37	2 (5.4)
	Lesser noddy	*Anous tenuirostris*	Mar. 2013	Nonbreeding adults	57	45 (78.9)
Tromelin	Masked booby	*Sula dactylatra*	Sep. 2012	Breeding adults and chicks	22	1 (4.5)
	Red-footed boody	*Sula sula*	Sep. 2012	Breeding adults and chicks	21	0
