# Supplementary material for: Influenza A Virus on Oceanic Islands: Host and Viral Diversity in Seabirds in the Western Indian Ocean
Source: PLoS Pathog. 2015 May 21;11(5):e1004925. doi: 10.1371/journal.ppat.1004925 (PMC4440776; doi:10.1371/journal.ppat.1004925)
Supplement: S3 Table — (PDF) [file ppat.1004925.s003.pdf]

Island	Species		Date	Status	N tested	N Positive
Aride	Brown noddy	*Anous stolidus*	Jun. 2012	Breeding adults	37 (CL)	0
	Lesser noddy	*Anous tenuirostris*	Jun. 2012	Breeding adults	50 (CL)	0
	Sooty tern	*Onychoprion fuscatus*	Jun. 2012	Breeding adults	101 (CL)	0
Bird	Brown noddy	*Anous stolidus*	Jun. 2012	Breeding adults	33 (CL)	0
	Lesser noddy	*Anous tenuirostris*	Jun. 2012	Breeding adults	32 (CL)	0
	Sooty tern	*Onychoprion fuscatus*	Jun. 2012	Breeding adults	93 (CL)	0
	Brown noddy	*Anous stolidus*	Jun. 2013	Breeding adults	90 (CL)	0
	Lesser noddy	*Anous tenuirostris*	Jun. 2013	Breeding adults	90 (CL)	0
	Sooty tern	*Onychoprion fuscatus*	Jun. 2013	Breeding adults	100 (CL)	0
Europa	Sooty tern	*Onychoprion fuscatus*	Jul. 2012	Breeding adults	194 (CL)	0
	Sooty tern	*Onychoprion fuscatus*	Nov. 2012	Breeding adults and chicks	82 (CL) + 81 (OP)	0
Juan de Nova	Sooty tern	*Onychoprion fuscatus*	Dec. 2012	Breeding adults and chicks	126 (CL) + 125 (OP)	0
Reunion	Lesser noddy	*Anous tenuirostris*	Mar. 2013	Nonbreeding adults	58 (CL) + 58 (OP)	14 (CL) + 3 (OP)
