# Supplementary figures and images for: Influenza A Virus on Oceanic Islands: Host and Viral Diversity in Seabirds in the Western Indian Ocean
Source: PLoS Pathog. 2015 May 21;11(5):e1004925. doi: 10.1371/journal.ppat.1004925 (PMC4440776; doi:10.1371/journal.ppat.1004925)

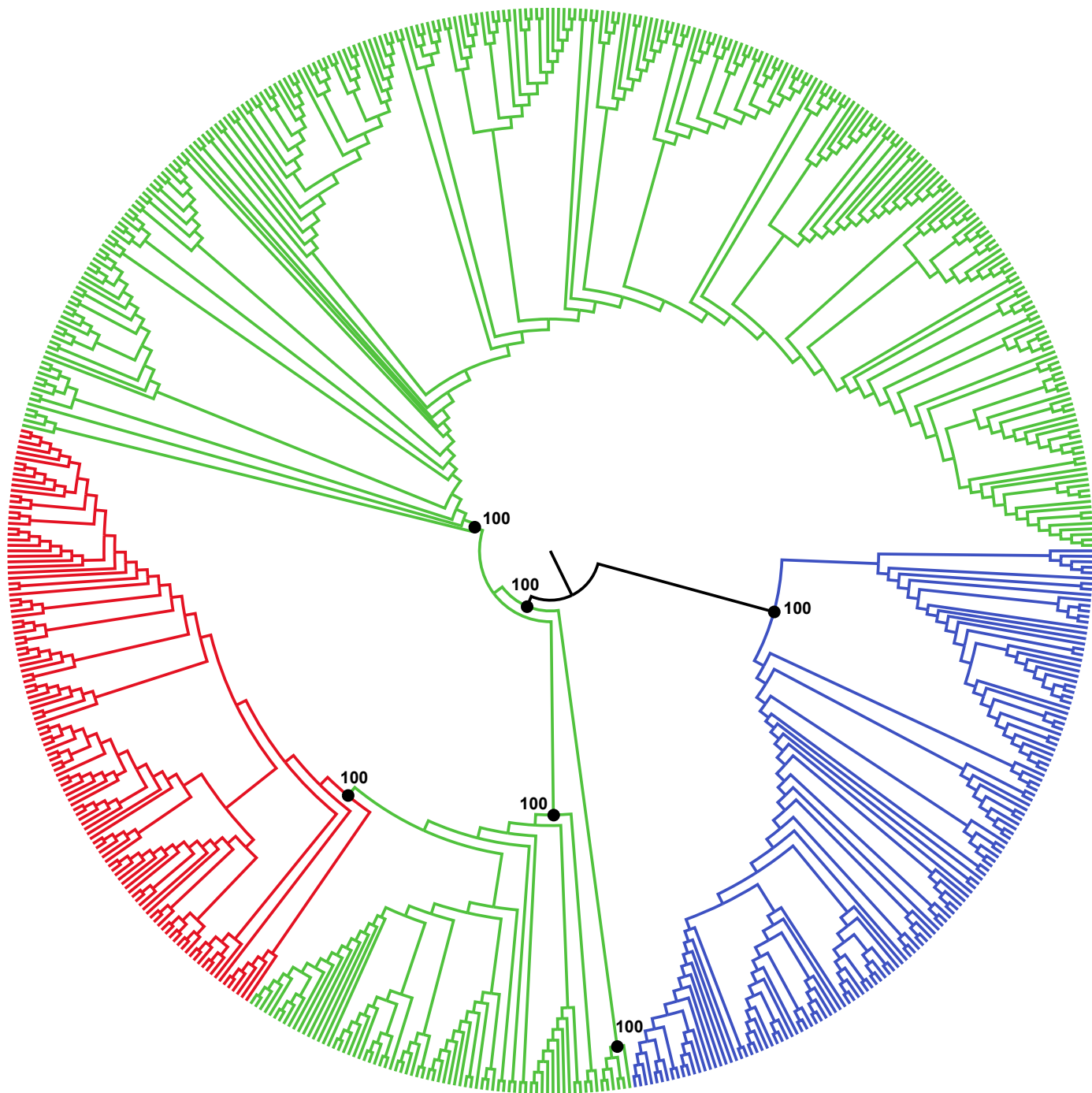

Supplement: S1 Fig — Computations were realized with the GTR+I+ Γ evolutionary model (I = 0.3; α = 1.3). Blue branches highlight viruses isolated in humans and green branches viruses recovered from birds, swines and from the environment. Red branches highlight the genetic lineage of Reunion Island H2 influenza A viruses for which the detailed evolutionary history was investigated with coalescent analyses (Fig 4). Bootstrap values are indicated for the main phylogenetic lineages (black circles). (PDF) [file ppat.1004925.s005.pdf]
